# Ups and downs of fossorial life: migration restlessness and geotaxis may explain overwintering emergence in the spotted salamander

**DOI:** 10.1242/jeb.249319

**Published:** 2024-10-24

**Authors:** Danilo Giacometti, Patrick D. Moldowan, Glenn J. Tattersall

**Affiliations:** ^1^ Department of Biological Sciences, Brock University, St Catharines, ON, Canada, L2S 3A1; ^2^ Department of Ecology and Evolutionary Biology, University of Toronto, Toronto, ON, Canada, M5S 1A4; ^3^ School of the Environment, University of Toronto, Toronto, ON, Canada, M5S 1A4; ^4^ Algonquin Wildlife Research Station, Whitney, ON, Canada, K0J 2M0

**Keywords:** Amphibian, Behaviour, Ectotherm, Gravity, Migration, Temperature

## Abstract

To decide whether to remain underground or to emerge from overwintering, fossorial ectotherms simultaneously process environmental, gravitational and circannual migratory cues. Here, we provide an experimental framework to study the behaviour of fossorial ectotherms during soil temperature inversion – a phenomenon that marks the transition between winter and spring – based on three non-mutually exclusive hypotheses (thermoregulation, negative geotaxis and migration restlessness). Using a vertical thermal gradient, we evaluated how temperature selection (*T*_sel_), activity and vertical position selection differed under simulated soil temperature inversion (contrasting the active versus overwintering thermal gradients) in the spotted salamander (*Ambystoma maculatum*). Salamanders had different *T*_sel_ and activity levels between gradients, but selected similar heights regardless of thermal gradient orientation. Negative geotaxis may explain responses to changes in vertical thermal gradient orientation, with migratory restlessness contributing to differences in activity levels. Ultimately, our work should benefit those who aim to better understand the biology of fossorial ectotherms.

## Teaser

**Summary:** Analysis of *Ambystoma maculatum* during overwintering emergence shows that both innate (migration restlessness) and external (temperature and gravity) factors can explain the behaviour of fossorial ectotherms emerging from underground burrows at the end of winter.

## INTRODUCTION

Fossorial animals are considered to be buffered from extreme temperature fluctuations because underground temperatures are generally more stable than those aboveground (Kinlaw, 1999; Scheffers et al., 2014). While this rationale may apply in the short term (e.g. hours), underground temperatures still vary considerably over longer time scales (e.g. months) (Alves and Schmid, 2015; Cui et al., 2011). In the temperate zone, the transition between winter and spring is marked by a major shift in the temperature gradient between shallow and deep soils, a phenomenon known as soil temperature inversion (Moldowan et al., 2022). In the winter, shallow depths are colder than deep ones, but as spring arrives, the gradient shifts such that shallow depths become warmer than deep ones (Cui et al., 2011; Moldowan et al., 2022) (Fig. S1). Fossorial ectotherms may therefore use shifts in soil temperature as cues to emerge from overwintering (Milsom and Jackson, 2011; Ultsch, 1989). The spotted salamander (*Ambystoma maculatum*) provides a notable example. Coincident with spring rainfall, rapid snow melt and possibly soil temperature inversion, salamanders emerge from underground and migrate toward bodies of water to breed (Sexton et al., 1990). After a brief breeding period, individuals return to their burrows and only venture aboveground to forage when environmental conditions are appropriate (Moldowan et al., 2022; Sexton, 1986). Despite considerable effort in studying fossorial ectotherms before and after overwintering (e.g. Madison, 1997; Oldham, 1966), there is still limited knowledge about underground behaviours and environmental triggers involved in post-overwintering emergence (Ruibal et al., 1969).

In Amphibia, approximately 11% of known species (723 out of ∼6600 species) are fossorial (Oliveira et al., 2017). Despite this, the cryptic nature and abbreviated periods of aboveground activity of fossorial amphibians have generally hampered research in these species (Jared et al., 1999). As a result, our knowledge about fossorial amphibians is still incipient, and basic assumptions about their biology remain to be tested (Giacometti and Tattersall, 2023). Recently, radio telemetry work in Fowler's toad (*Anaxyrus fowleri*) showed that individuals moved vertically along underground thermal gradients to thermoregulate during summer months (Forget-Klein and Green, 2021). By adjusting the depth to which they were buried, *A*. *fowleri* maintained body temperature within their known range of selected temperatures (Brattstrom, 1963; Forget-Klein and Green, 2021). The ability to move vertically along underground thermal gradients presupposes the assimilation of at least three different sensory inputs: soil temperature, soil humidity and gravity. Responses to soil temperature and humidity are complementary and have been the focus of extensive research, as amphibians balance thermoregulation and hydroregulation to ensure proper physiological functioning (Brattstrom, 1979; Spotila, 1972). By contrast, evidence for gravity effects over amphibians is mixed. For example, gravity was shown to incur locomotory costs to salamanders moving on vertical but not horizontal surfaces (Aretz et al., 2022), but geotaxis (i.e. gravity-mediated movement) did not explain movement patterns in frogs and toads (Landreth and Ferguson, 1967; Oldham, 1966). Although evidence suggests that geotactic responses depend on soil temperature and humidity (Hoffman and Katz, 1989; Székely et al., 2018), it is unclear how these factors interplay to affect the behaviour of fossorial amphibians during soil temperature inversion.

Besides providing a framework to assess the effect of contrasting abiotic factors on behaviour, the context of soil temperature inversion also allows us to address behavioural responses to endogenous aspects. Migration restlessness, or ‘Zugunruhe’, is a circannual phenomenon characterised by an increase in activity levels and directional orientation (Gwinner, 1977). Although primarily studied in birds, migration restlessness has also been suggested to occur in marine turtles, as evidenced by hyperactivity and an increase in surfacing behaviour (Mansfield et al., 2009), and in salamanders, through hormone-mediated increases in locomotor activity (Gona et al., 1973). From a behavioural standpoint, amphibian migration has been studied through the lens of homing, orientation or dispersal capacity (Jreidini and Green, 2022; Sinsch, 1991, 2006). As such, it is unclear whether migration restlessness is a widespread behaviour across amphibians. Given that soil temperature inversion may prompt fossorial amphibians to emerge from overwintering and migrate to breeding ponds, individuals should simultaneously process thermal, gravitational and endogenous migratory cues to decide whether to remain underground or move to the surface. Improper integration of these cues could ultimately result in amphibians partaking in overland migration too early in the year, thus risking freezing (Madison, 1997; Moldowan et al., 2022).

Here, we tested three non-mutually exclusive hypotheses that explain behavioural responses to soil temperature inversion in *A*. *maculatum*. Using a vertical thermal gradient in a laboratory, we evaluated how temperature selection (*T*_sel_), vertical position (i.e. height within the thermal gradient) and activity differed under simulated soil temperature inversion (i.e. active versus overwintering thermal gradient orientation) while controlling for relative humidity. The ‘thermoregulatory hypothesis’ suggests that temperature is the main factor influencing behaviour, and salamanders should select temperatures within their known winter *T*_sel_ range (Giacometti and Tattersall, 2024) regardless of thermal gradient orientation. The ‘negative geotaxis hypothesis’ posits that salamanders should position themselves at locations above the mid-point of the vertical thermal gradient (i.e. move or stay higher up, rather than down) regardless of thermal gradient orientation, as amphibians burrow near the surface under high humidity conditions (Székely et al., 2018). The ‘migration restlessness hypothesis’ suggests that thermal gradient inversion provides a signal for the onset of breeding migration, leading to differences in directional vertical movements and activity levels between thermal gradient orientations (Mansfield et al., 2009). The predicted outcomes of our variables of interest under each hypothesis are detailed in Table 1. By testing these hypotheses, we aim to disentangle the effect of temperature and gravity on salamander behaviour, ultimately furthering the knowledge about underground behaviour and behavioural responses to post-overwintering emergence in fossorial amphibians.

**Table 1.**
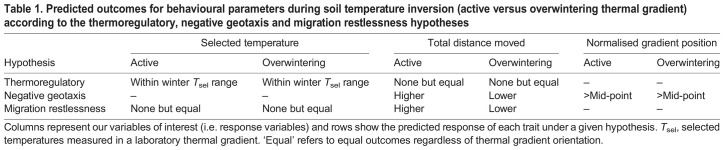
Predicted outcomes for behavioural parameters during soil temperature inversion (active versus overwintering thermal gradient) according to the thermoregulatory, negative geotaxis and migration restlessness hypotheses

## MATERIALS AND METHODS

### Study species and husbandry

*Ambystoma maculatum* (Shaw 1802) is a species of mole salamander widely distributed in eastern North America (O'Donnell, 1937). While the subterranean life of *A*. *maculatum* is poorly known, evidence suggests that individuals occupy mammal burrows 20–75 cm below ground level, with horizontal burrows being used in the spring and summer, and more vertical burrows being used in the winter (Madison, 1997; Regosin et al., 2003; Windmiller, 1996). In May 2022, we collected adult *A*. *maculatum* using a drift fence installed around Bat Lake, Algonquin Provincial Park, ON, Canada (45.5857°N, 78.5185°W) with authorisation from Brock University's Animal Care Committee (AUP #22-03-04), the Ministry of Northern Development, Mines, Natural Resources and Forestry (#1100575), and Ontario Parks. To transfer the salamanders from Bat Lake to Brock University, we placed them in a plastic container with a ventilated fitted lid (34 cm×19.6 cm×12 cm L×W×H). The container had *Sphagnum* moss, pine needles and water, and was placed inside a transport box kept at ∼4°C to avoid overheating and dehydration during transportation.

In the lab, we housed salamanders in pairs within ventilated tanks that contained wet coconut husk fibre, *Sphagnum* moss and PVC pipe refuges; tanks were misted regularly to maintain high humidity. These tanks were kept in a facility with controlled temperature, relative humidity (RH) and photoperiod. We adjusted temperature and photoperiod seasonally to mimic Bat Lake conditions (Moldowan et al., 2022) while always maintaining RH at 70%. Individual salamanders were identified by their unique spot pattern. We fed salamanders twice a week with mealworms dusted in calcium and multivitamin powder; water was available *ad libitum*. The current study was conducted between March and April 2024 using animals that had been acclimatised to winter conditions (2°C and under total darkness) for at least 4 weeks and were not fed during this time.

### Laboratory thermal gradient

To assess the effect of temperature and gravity on salamander behaviour, we used a rectangular thermal gradient (54 cm×25 cm×3 cm L×W×H) built by Brock University Technical Services (Fig. S2; Movie 1). The thermal gradient was mounted on a metal cradle, allowing us to adjust its orientation from completely horizontal to completely vertical (i.e. from 0 to 90 deg). Based on information on the angle of burrows (Seabloom et al., 2000; Vleck, 1981), we always kept our thermal gradient at 45 deg relative to the horizontal axis. The copper floor of the thermal gradient had copper pipes that were connected to hot- and cold-recirculating water baths (Haake™, models DC10 and SC100, respectively), creating a near-linear temperature gradient ranging from approximately −1.5 to 15°C (Fig. S3), measured to the nearest 0.01°C with a thermocouple meter (Sable Systems, model TC-1000). We chose this temperature range because it represents possible temperatures experienced by *A*. *maculatum* in Bat Lake during soil temperature inversion (Fig. S1) (Moldowan et al., 2022). To simulate soil temperature inversion, we alternated whether warm or cold water would be delivered to the top or bottom of the gradient. We refer to ‘active thermal gradient’ as the thermal gradient orientation experienced by salamanders during their normal active season (i.e. warm top and cold bottom) (Fig. S2B). Conversely, we refer to ‘overwintering thermal gradient’ as the thermal gradient experienced by salamanders during the overwintering season (i.e. cold top and warm bottom) (Fig. S2C).

To mimic the tunnel-like nature of burrows, we placed four sponges (14 cm×4 cm×3 cm L×W×H) equidistantly every 3 cm on either side of the thermal gradient along its longest wall (Fig. S2). To ensure the sponges remained in place during the experiments, we sewed the hook side of Velcro tape onto the sponges and used aquarium safe silicon sealant (Adhesive Guru, product AG310) to attach the loop side of the Velcro tape to the floor of the thermal gradient. Before each experiment, we submerged the sponges in 1 l of dechlorinated water for at least 5 h. We then attached the water-soaked sponges to the floor of the thermal gradient so that salamanders had a source of humidity at all gradient temperatures once the thermal gradient lid was closed; we verified this with a RH logger (Inkbird, model IBS-TH1) (mean±s.d.; 89.88±6.53%). The lid of the thermal gradient was made of transparent anti-glare material, allowing us to remotely view animals at all times during the experiments. To prevent inadvertent building vibrations from disturbing the animals or altering the angle of the thermal gradient, we placed the thermal gradient on top of 15 cm of insulation foam board and padding. We conducted our experiments in total darkness to match conditions experienced by *A*. *maculatum* within burrows. Two infrared illuminators (wavelength 850 nm; TVPSii, model TP-IRBP15) positioned 200 cm away and aiming diffuse light above the thermal gradient allowed us to continuously visualise the animals through a livestream. We recorded the salamanders with a high-resolution infrared webcam (Agama, model V-1325R) positioned 130 cm in front of the centre of the thermal gradient. This webcam was connected to time-lapse image acquisition software (HandyAVI^®^) set to capture an image every 30 s.

### Experimental design

We allowed the salamanders (*N*=15; 7 females and 8 males) a total of 18 h inside the thermal gradient, from ∼16:00 h to 10:00 h. Each animal was tested twice under each thermal gradient orientation in random order (*N*=30 trials total), with a minimum interval of 7 days between trials. We gave the salamanders an initial 3 h habituation period, and used the data obtained in the subsequent 15 h in the analyses. We always allowed a minimum interval of 6 h between trials after disinfecting the stage of the thermal gradient with 70% ethanol at the end of a trial.

We always handled the salamanders using nitrile gloves. Before introducing a salamander into the thermal gradient, we placed it into a container filled with 30 ml of dechlorinated water for 15 min so it could absorb water through its skin. We then weighed the salamander and placed it into the thermal gradient, determining at random whether the salamander would be initially facing the wall or the centre of the gradient. We always introduced the salamanders into the gradient at the 2°C point to match their acclimatisation temperature. After finishing an experiment, we removed the salamander from the thermal gradient and weighed it. We used the difference in body mass before and after an experiment divided by the length of our experiment as an indirect measure of evaporative water loss (EWL; g h^−1^) (Navas and Araujo, 2000). After weighing, we placed the salamander back in its housing tank.

### Data processing

We captured a total of 2160 images for each individual over the course of each 18 h long experiment. We imported image sequences into Fiji (Schindelin et al., 2012), and recorded the identity of the individual (ID), the thermal gradient orientation (active versus overwintering) and the sex of the individual. For each image sequence, we used the manual tracking plug-in in Fiji to track the location of the mid-body of the salamander within the thermal gradient. The output of the manual tracking function was a set of Cartesian (*x*,*y*) coordinates that had *x*,*y*=0,0 as the top left of the image. We then converted salamander body position into selected gradient temperature following Giacometti et al. (2021). Importantly, we assumed that the body temperature of the salamanders was in thermal equilibrium with the thermal gradient floor based on research showing that *T*_sel_ matched core temperatures over long time courses (Cadena and Tattersall, 2009). Because our study aimed at disentangling the effect of temperature and gravity on salamander behaviour rather than measuring thermoregulation in *A*. *maculatum* (e.g. thermoregulatory precision and accuracy), we only calculated median *T*_sel_ instead of the parameters that make up the dual set-point concepts of thermoregulation in ectotherms (Barber and Crawford, 1977).

To obtain a measure of activity within the thermal gradient, we calculated the distance moved by each salamander (*d_t_*) every 30 s using:
(1)

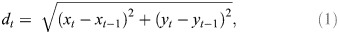
where *x* and *y* are cartesian coordinates converted into distance (metres), and *t* and *t*–1 are adjacent time points for all *n* time points. Total distance moved was the sum of *d_t_* for each individual:
(2)

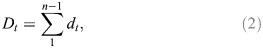
where *n* is the total number of images (*n*=2160). To estimate geotaxis, we converted salamander position along the *y*-axis (i.e. vertical position) into an index of normalised gradient position (*y*_norm_):
(3)

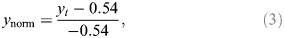
where *y_t_* is a cartesian coordinate converted into distance (metres) at time point *t*, and 0.54 is the length of the thermal gradient in metres. We used a negative denominator so that *y*_norm_ became an index that ranged from 0 to 1. Thus, if *y*_norm_ is close to 0, then animals positioned themselves near the bottom of the thermal gradient (i.e. positive geotaxis). If *y*_norm_ is close to 1, then animals positioned themselves near the top of the thermal gradient (i.e. negative geotaxis).

### Data analysis

We performed all analyses using R (version 4.3.2) in RStudio (version 2024.04.0) (http://www.R-project.org/) assuming a significance level of 0.05. To search for deviations from normality and homoscedasticity, we visually inspected *Q*–*Q* and *P*–*P* plots with the ‘fitdist’ function from the fitdistrplus package (Delignette-Muller and Dutang, 2015). To test our hypotheses, we built linear mixed-effects models (LMMs) with the ‘lmer’ function from the lme4 package set to default parameters (Bates et al., 2015). We considered median *T*_sel_, total distance moved and normalised gradient position as response variables in our models. We fitted one model per response variable, including gradient orientation (categorical; active versus overwintering), sex (categorical; male or female) and final body mass (continuous) as fixed terms in all models. To account for multiple observations per individual, we also included salamander ID as a random term in our models. To compare whether individual normalised gradient position differed from the initial position animals were placed in the thermal gradient (active: μ=0.25; overwintering: μ=0.75), we fitted a one-sample *t*-test with the ‘t.test’ function from the stats package (http://www.R-project.org/). We assessed residual autocorrelation with the ‘checkresiduals’ function from the forecast package (https://CRAN.R-project.org/package=forecast), and the ‘qqnorm’ and ‘acf’ functions from the stats package (http://www.R-project.org/). We evaluated model fit using the ‘check_model’ function from the performance package (Lüdecke et al., 2021) and visualised fixed model effects by plotting an object created with the ‘allEffects’ function from the effects package (https://CRAN.R-project.org/package=effects). We created figures using the ggplot2 (Wickham, 2016), Thermimage (https://CRAN.R-project.org/package=Thermimage) and cowplot (https://CRAN.R-project.org/package=cowplot) packages. Our data and code can be accessed from Brock University Dataverse: https://doi.org/10.5683/SP3/NBJYTM.

## RESULTS AND DISCUSSION

**Fig. 1.**
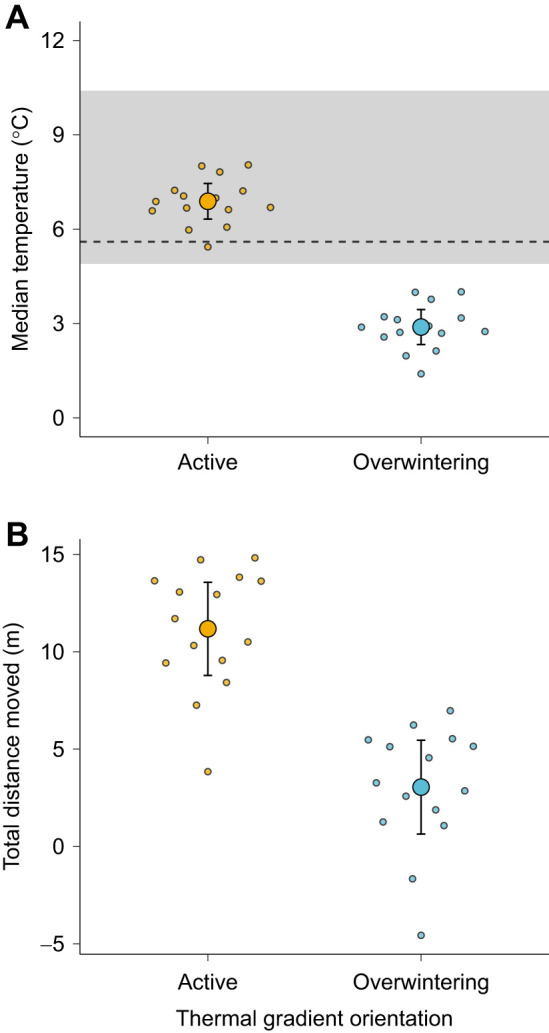
**Effect of thermal gradient orientation on selected temperatures and distance moved of *Ambystoma maculatum*.** (A) Differences in median selected gradient temperature between salamanders tested in the active and overwintering thermal gradients. The grey shaded area represents the known range of selected temperatures in overwintering *A*. *maculatum*. The dashed line indicates the approximate mid-point temperature of the thermal gradients used in the study. (B) Differences in total distance moved between salamanders tested in the active and overwintering thermal gradients. In both panels, large circles represent the mean predicted value of a given trait for all salamanders, and bars indicate the corresponding 95% confidence intervals. Small circles represent the mean predicted value of a given trait for each individual salamander.

Thermal gradient orientation affected *T*_sel_ (Table S1), with salamanders selecting higher temperatures in the active compared with the overwintering thermal gradient (active *T*_sel_=6.89±2.84°C; overwintering *T*_sel_=2.89±1.06°C) (Fig. 1A). In the active thermal gradient, *T*_sel_ matched values previously measured in *A*. *maculatum* during the winter (Giacometti and Tattersall, 2024). In the overwintering thermal gradient, however, *T*_sel_ values were lower than the known range of temperatures selected by *A*. *maculatum* in the winter (Giacometti and Tattersall, 2024). Salamanders had similar rates of EWL between gradients (active EWL=0.04±0.42 ml h^−1^; overwintering EWL=0.03±0.49 ml h^−1^), indicating that water balance constraints did not impact *T*_sel_ (Brattstrom, 1979). Thus, these results suggest that thermoregulation was not the primary factor behind the observed behavioural differences between gradients. While some fossorial ectotherms actively thermoregulate (Forget-Klein and Green, 2021; Wu et al., 2009), it is argued that these species have blunted thermal sensitivities that translate into lower thermophily compared with non-fossorial ectotherms (Camacho et al., 2015). Recent research showed that *A*. *maculatum* exhibits a 9.4°C shift in median *T*_sel_ between the active (17.0±1.77°C) and overwintering (7.60±2.10°C) seasons, highlighting that this species can remain active across a wide temperature range (Giacometti and Tattersall, 2024). This heightened plasticity of thermal biology could be important during overwintering emergence, as *A*. *maculatum* may be able to occupy relatively shallow burrows (i.e. relatively cool) despite potentially high thermal variability in depths up to 50 cm (Moldowan et al., 2022). If this is the case, then salamanders would be able to respond to soil temperature inversion quicker than if they were in deeper burrows, thereby maximising near-surface foraging (Gordon, 1968) and potentially prolonging their breeding season; these hypotheses remain to be tested. Additionally, if migrating salamanders encounter unfavourable conditions aboveground before reaching breeding pools, they should be able to retreat to shallow mammal runways until environmental conditions improve (Madison, 1997).

**Fig. 2.**
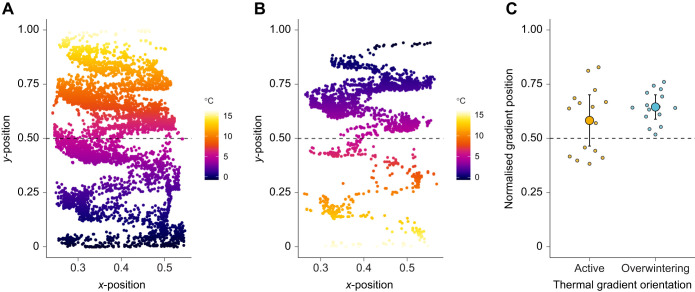
**Effect of thermal gradient orientation on salamander distribution and height selection.** (A,B) Distribution of *A. maculatum* within the active (A) and overwintering (B) thermal gradients. Each circle represents the position of an individual salamander over the course of 18 h within a thermal gradient (colour coded). (C) Comparison of normalised gradient position between *A*. *maculatum* tested in the active and overwintering thermal gradients. Large circles represent the mean predicted gradient position for all salamanders, and bars indicate the corresponding 95% confidence intervals. Small circles represent the mean predicted gradient position of each individual salamander. In all panels, the dashed line represents the mid-point of the thermal gradient.

Salamanders moved greater distances in the active relative to the overwintering thermal gradient (active *D_t_*=11.20±12.50 m; overwintering *D_t_*=3.05±6.37 m) (Fig. 1B; Table S2), as posited by the migration restlessness hypothesis. This difference in activity between gradients also reflects the finding that salamanders explored all available temperatures in the active but not in the overwintering thermal gradient (Fig. 2A,B). From a sensory perspective, decreased movement in the overwintering thermal gradient could be interpreted as salamanders perceiving the direction of thermal stimuli in the gradient (i.e. cold top and warm bottom) through thermosensitive units in the skin (Hutchison and Dupré, 1992) and limiting activity to save energy (Holden et al., 2021). Although studies on the thermosensitivity of amphibian skin are relatively scarce, evidence suggests that cold receptors are more responsive to thermal stimuli than warm receptors, especially in the rostrum (Spray, 1974, 1986). Given that behavioural responses in ectotherms are fundamentally physiologically constrained (Sakich et al., 2023), future work may explore the relationship between rostral thermosensation (Black and Tattersall, 2017) and locomotion (Inoue et al., 2014) during thermal gradient inversion to clarify the factors that impact locomotion in amphibians that overwinter underground.

Interestingly, the values we report for total distance moved for both gradient orientations are much lower than those measured in *A*. *maculatum* over a 9 h period in a horizontal thermal gradient during the active (109.0±67.10 m) and overwintering (78.20±70.50 m) seasons (Giacometti and Tattersall, 2024). While thermal gradient design (e.g. length, shape) could affect how much an ectotherm moves (Dillon et al., 2012), the low distances we found could be better explained through gravity imposing biomechanical costs on locomotion. For instance, previous work demonstrated that gravity limits locomotion through changes in the centre of gravity, gait and velocity of salamanders travelling up or down vertical surfaces (Aretz et al., 2022; Hanna et al., 2022). Indeed, movements along the vertical axis may be so costly that salamanders may opt to jump instead of climb down a vertical surface (Aretz et al., 2022). Preliminary evidence suggests that angles between 60 and 90 deg do not impact vertical movements in arboreal salamanders (Aretz et al., 2022); however, cling performance differs substantially between arboreal and fossorial salamanders. For example, the arboreal *Bolitoglossa franklini* had a maximum cling angle of 174±4 deg compared with 99±5 deg in the fossorial *A*. *maculatum* (Baken and O'Donnell, 2021). To better understand the overwintering ecology of fossorial amphibians, future work may assess how altering the angle of vertically oriented thermal gradients impacts locomotion in species that only climb periodically.

Thermal gradient orientation did not affect vertical position in *A*. *maculatum*, as evidenced by salamanders occupying similar heights in the two orientations (Fig. 2C; Table S3). Salamanders consistently occupied positions at or above the mid-point of the gradients (active: *t*_14_=8.38, *P*<0.001; overwintering: *t*_14_=−5.58, *P*<0.001), indicating the presence of negative geotactic (i.e. movement against the force of gravity) responses in *A*. *maculatum*. Coupled with our data on total distance moved, these results suggest that salamanders were willing to pay the cost of climbing to stay near the top of the thermal gradient, regardless of its orientation. While geotactic responses in amphibians still warrant further research, our results are backed up by studies demonstrating negative geotaxis in newts and toads (Cummings, 1912; FitzGerald and Bider, 1974). Importantly, the strength of negative geotaxis may differ between seasons, which indicates that geotactic responses may be context dependent (Cummings, 1912). In the current study, we studied behaviours associated with post-overwintering emergence (late winter/early spring), which coincides with the time when *A*. *maculatum* leave their burrows and partake in overland migration (Sexton, 1986). To test the consistency of negative geotaxis in *A*. *maculatum*, future work may study salamanders at the start of the overwintering season (i.e. late autumn/early winter), which is the period when *A*. *maculatum* shifts from using horizontal to vertical burrows in preparation for winter (Madison, 1997).

### Conclusions

By testing three non-mutually exclusive hypotheses, we showed that negative geotaxis best explained how *A*. *maculatum* responded to changes in vertical thermal gradient orientation, with a potential role for migratory restlessness contributing to differences in activity levels (Table 1). This conclusion is supported by the findings that salamanders (i) selected temperatures below their winter *T*_sel_ in the overwintering thermal gradient, (ii) moved greater distances in the active relative to the overwintering thermal gradient, and (iii) selected gradient heights at or above the mid-point of the thermal gradient regardless of its orientation. Our study contributes to the goal of elucidating the physiology and behaviour of fossorial amphibians, which are understudied relative to their non-fossorial counterparts (Giacometti and Tattersall, 2023; Jared et al., 1999; Székely et al., 2018). Future work may build on our findings, and investigate how other sensory stimuli (e.g. acoustic, olfactory) (Sinsch, 1991) impact amphibian behaviour in ecologically relevant scenarios.

## Supplementary Material

10.1242/jexbio.249319_sup1 Supplementary information

## References

[JEB249319C1] Alves, A. B. M. and Schmid, A. L. (2015). Cooling and heating potential of underground soil according to depth and soil surface treatment in the Brazilian climatic regions. *Energy Build*. 90, 41-50. 10.1016/j.enbuild.2014.12.025

[JEB249319C2] Aretz, J. M., Brown, C. E. and Deban, S. M. (2022). Vertical locomotion in the arboreal salamander *Aneides vagrans*. *J. Zool*. 316, 72-79. 10.1111/jzo.12934

[JEB249319C3] Baken, E. K. and O'Donnell, M. K. (2021). Clinging ability is related to particular aspects of foot morphology in salamanders. *Ecol. Evol*. 11, 11000-11008. 10.1002/ece3.7888 34429897 PMC8366850

[JEB249319C4] Barber, B. J. and Crawford, E. C. (1977). A stochastic dual-limit hypothesis for behavioral thermoregulation in lizards. *Physiol. Zool*. 50, 53-60. 10.1086/physzool.50.1.30155715

[JEB249319C5] Bates, D., Mächler, M., Bolker, B. and Walker, S. (2015). Fitting linear mixed-effects models using lme4. *J. Stat. Softw*. 67, 1-48. 10.18637/jss.v067.i01

[JEB249319C6] Black, I. R. G. and Tattersall, G. J. (2017). Thermoregulatory behavior and orientation preference in bearded dragons. *J. Therm. Biol*. 69, 171-177. 10.1016/j.jtherbio.2017.07.009 29037379

[JEB249319C7] Brattstrom, B. H. (1963). A preliminary review of the thermal requirements of amphibians. *Ecology* 44, 238-255. 10.2307/1932171

[JEB249319C8] Brattstrom, B. H. (1979). Amphibian temperature regulation studies in the field and laboratory. *Am. Zool*. 19, 345-356. 10.1093/icb/19.1.345

[JEB249319C9] Cadena, V. and Tattersall, G. J. (2009). The effect of thermal quality on the thermoregulatory behavior of the bearded dragon *Pogona vitticeps*: influences of methodological assessment. *Physiol. Biochem. Zool*. 82, 203-217. 10.1086/597483 19323642

[JEB249319C10] Camacho, A., Pavão, R., Moreira, C. N., Pinto, A. C. B. C. F., Navas, C. A. and Rodrigues, M. T. (2015). Interaction of morphology, thermal physiology and burrowing performance during the evolution of fossoriality in Gymnophthalmini lizards. *Funct. Ecol*. 29, 515-521. 10.1111/1365-2435.12355

[JEB249319C11] Cui, W., Liao, Q., Chang, G., Chen, G., Peng, Q. and Jen, T.-C. (2011). Measurement and prediction of undisturbed underground temperature distribution. Proceedings of the ASME 2011 International Mechanical Engineering Congress and Exposition, Volume 4: Energy Systems Analysis, Thermodynamics and Sustainability; Combustion Science and Engineering; Nanoengineering for Energy, Parts A and B. Denver, Colorado, USA, November 11–17, 2011, pp. 671-676. ASME. 10.1115/IMECE2011-63311

[JEB249319C12] Cummings, B. F. (1912). Distant orientation in Amphibia. *Proc. Zool. Soc. London*. 82, 8-19. 10.1111/j.1469-7998.1912.tb07000.x

[JEB249319C13] Delignette-Muller, M. L. and Dutang, C. (2015). fitdistrplus: An R package for fitting distributions. *J. Stat. Softw*. 64, 1-34. 10.18637/jss.v064.i04

[JEB249319C14] Dillon, M. E., Liu, R., Wang, G. and Huey, R. B. (2012). Disentangling thermal preference and the thermal dependence of movement in ectotherms. *J. Therm. Biol*. 37, 631-639. 10.1016/j.jtherbio.2012.07.004

[JEB249319C15] FitzGerald, G. J. and Bider, J. R. (1974). Evidence for a relationship between geotaxis and seasonal movements in the toad *Bufo americanus*. *Oecologia* 17, 277-280. 10.1007/BF00344927 28308172

[JEB249319C16] Forget-Klein, É. and Green, D. M. (2021). Toads use the subsurface thermal gradient for temperature regulation underground. *J. Therm. Biol*. 99, 102956. 10.1016/j.jtherbio.2021.102956 34420612

[JEB249319C17] Giacometti, D. and Tattersall, G. J. (2023). Putting the energetic-savings hypothesis underground: fossoriality does not affect metabolic rates in amphibians. *Evol. Ecol*. 37, 761-777. 10.1007/s10682-023-10253-5

[JEB249319C18] Giacometti, D. and Tattersall, G. J. (2024). Seasonal variation of behavioural thermoregulation in a fossorial salamander (*Ambystoma maculatum*). *R. Soc. Open Sci*. 11, 240537. 10.1098/rsos.240537 39233724 PMC11371426

[JEB249319C19] Giacometti, D., Yagi, K. T., Abney, C. R., Jung, M. P. and Tattersall, G. J. (2021). Staying warm is not always the norm: behavioural differences in thermoregulation of two snake species. *Can. J. Zool*. 99, 974-983. 10.1139/cjz-2021-0135

[JEB249319C20] Gona, A. G., Pearlman, T. and Gona, O. (1973). Effects of prolactin and thyroxine in hypophysectomized and thyroidectomized red efts of the newt *Notophthalmus* (*Diemictylus*) *viridescens*. *Gen. Comp. Endocrinol*. 20, 107-111. 10.1016/0016-6480(73)90135-4 4689577

[JEB249319C21] Gordon, R. E. (1968). Terrestrial activity of the spotted salamander, *Ambystoma maculatum*. *Copeia* 1968, 879-880. 10.2307/1441868

[JEB249319C22] Gwinner, E. (1977). Circannual rhythms in bird migration. *Annu. Rev. Ecol. Syst*. 8, 381-405. 10.1146/annurev.es.08.110177.002121

[JEB249319C23] Hanna, C. S., Alihosseini, C., Fischer, H. M., Davoli, E. C. and Granatosky, M. C. (2022). Are they arboreal? Climbing abilities and mechanics in the red-backed salamander (*Plethodon cinereus*). *J. Exp. Zool. A Ecol. Integr. Physiol.* 337, 238-249. 10.1002/jez.2561 34752693

[JEB249319C24] Hoffman, J. and Katz, U. (1989). The ecological significance of burrowing behaviour in the toad (*Bufo viridis*). *Oecologia* 81, 510-513. 10.1007/BF00378961 28312646

[JEB249319C25] Holden, K. G., Gangloff, E. J., Gomez-Mancillas, E., Hagerty, K. and Bronikowski, A. M. (2021). Surviving winter: Physiological regulation of energy balance in a temperate ectotherm entering and exiting brumation. *Gen. Comp. Endocrinol*. 307, 113758. 10.1016/j.ygcen.2021.113758 33771532

[JEB249319C26] Hutchison, V. H. and Dupré, R. K. (1992). Thermoregulation. In *Environmental Physiology of the Amphibians* (ed. M. E. Feder and W. W. Burggren), pp. 206-249. Chicago: University of Chicago Press.

[JEB249319C27] Inoue, T., Yamashita, T. and Agata, K. (2014). Thermosensory signaling by TRPM is processed by brain serotonergic neurons to produce planarian thermotaxis. *J. Neurosci*. 34, 15701-15714. 10.1523/JNEUROSCI.5379-13.2014 25411498 PMC6608440

[JEB249319C28] Jared, C., Navas, C. A. and Toledo, R. C. (1999). An appreciation of the physiology and morphology of the Caecilians (Amphibia: Gymnophiona). *Comp. Biochem. Physiol. A Mol. Integr. Physiol.* 123, 313-328. 10.1016/S1095-6433(99)00076-8

[JEB249319C29] Jreidini, N. and Green, D. M. (2022). Dispersal without drivers: Intrinsic and extrinsic variables have no impact on movement distances in a terrestrial amphibian. *Ecol. Evol*. 12, e9368. 10.1002/ece3.9368 36203625 PMC9526034

[JEB249319C30] Kinlaw, A. (1999). A review of burrowing by semi-fossorial vertebrates in arid environments. *J. Arid Environ*. 41, 127-145. 10.1006/jare.1998.0476

[JEB249319C31] Landreth, H. F. and Ferguson, D. E. (1967). Movements and orientation of the tailed frog, *Ascaphus truei*. *Herpetol* 23, 81-93.

[JEB249319C32] Lüdecke, D., Ben-Shachar, M. S., Patil, I., Waggoner, P. and Makowski, D. (2021). performance: An R package for assessment, comparison and testing of statistical models. *J. Open Source Softw*. 6, 31-39.

[JEB249319C33] Madison, D. M. (1997). The emigration of radio-implanted Spotted Salamanders, *Ambystoma maculatum*. *J. Herpetol*. 31, 542-551. 10.2307/1565607

[JEB249319C34] Mansfield, K. L., Saba, V. S., Keinath, J. A. and Musick, J. A. (2009). Satellite tracking reveals a dichotomy in migration strategies among juvenile loggerhead turtles in the Northwest Atlantic. *Mar. Biol*. 156, 2555-2570. 10.1007/s00227-009-1279-x

[JEB249319C35] Milsom, W. K. and Jackson, D. C. (2011). Hibernation and gas exchange. In *Comprehensive Physiology* (ed. R. Terjung), pp. 397-420. New Jersey: John Wiley & Sons, Inc. 10.1002/cphy.c090018 23737179

[JEB249319C36] Moldowan, P. D., Tattersall, G. J. and Rollinson, N. (2022). Climate-associated decline of body condition in a fossorial salamander. *Glob. Change Biol*. 28, 1725-1739. 10.1111/gcb.15766 34542922

[JEB249319C37] Navas, C. A. and Araujo, C. (2000). The use of agar models to study amphibian thermal ecology. *J. Herpetol*. 34, 330-334. 10.2307/1565438

[JEB249319C38] O'Donnell, D. J. (1937). Natural history of the ambystomid salamanders of Illinois. *Am. Midl. Nat*. 18, 1063-1071. 10.2307/2420604

[JEB249319C39] Oldham, R. S. (1966). Spring movement in the American toad, *Bufo americanus*. *Can. J. Zool*. 44, 63-100. 10.1139/z66-006

[JEB249319C40] Oliveira, B. F., São-Pedro, V. A., Santos-Barrera, G., Penone, C. and Costa, G. C. (2017). AmphiBIO, a global database for amphibian ecological traits. *Sci. Data* 4, 1-7. 10.1038/sdata.2017.123 PMC5584397 28872632

[JEB249319C41] Regosin, J. V., Windmiller, B. S. and Reed, J. M. (2003). Influence of abundance of small-mammal burrows and conspecifics on the density and distribution of spotted salamanders (*Ambystoma maculatum*) in terrestrial habitats. *Can. J. Zool*. 81, 596-605. 10.1139/z03-046

[JEB249319C42] Ruibal, R., Tevis, L. and Roig, V. (1969). The terrestrial ecology of the spadefoot toad *Scaphiopus hammondii*. *Copeia* 1969, 571-584. 10.2307/1441937

[JEB249319C43] Sakich, N. B., Bartel, P. C., Richards, M. H. and Tattersall, G. J. (2023). Hot crabs with bold choices: Temperature has little impact on behavioural repeatability in Caribbean hermit crabs. *Behav. Process*. 210, 104916. 10.1016/j.beproc.2023.104916 37454746

[JEB249319C44] Scheffers, B. R., Edwards, D. P., Diesmos, A., Williams, S. E. and Evans, T. A. (2014). Microhabitats reduce animal's exposure to climate extremes. *Glob. Change Biol*. 20, 495-503. 10.1111/gcb.12439 24132984

[JEB249319C45] Schindelin, J., Arganda-Carreras, I., Frise, E., Kaynig, V., Longair, M., Pietzsch, T., Preibisch, S., Rueden, C., Saalfeld, S., Schmid, B. et al. (2012). Fiji: an open-source platform for biological-image analysis. *Nat. Methods* 9, 676-682. 10.1038/nmeth.2019 22743772 PMC3855844

[JEB249319C46] Seabloom, E. W., Reichman, O. J. and Gabet, E. J. (2000). The effect of hillslope angle on pocket gopher (*Thomomys bottae*) burrow geometry. *Oecologia* 125, 26-34. 10.1007/PL00008888 28308219

[JEB249319C47] Sexton, O. J. (1986). Field studies of breeding spotted salamanders, *Ambystoma maculatum*, in eastern Missouri, USA. *Contrib. .Biol. Geol*. 67, 4-21.

[JEB249319C48] Sexton, O. J., Phillips, C. and Bramble, J. E. (1990). The effects of temperature and precipitation on the breeding migration of the spotted salamander (*Ambystoma maculatum*). *Copeia* 1990, 781-787.

[JEB249319C49] Sinsch, U. (1991). Mini-review: the orientation behaviour of amphibians. *Herpetol. J*. 1, 541-544.

[JEB249319C50] Sinsch, U. (2006). Orientation and navigation in Amphibia. *Mar. Freshw. Behav. Physiol*. 39, 65-71. 10.1080/10236240600562794

[JEB249319C51] Spotila, J. R. (1972). Role of temperature and water in the ecology of lungless salamanders. *Ecol. Monogr*. 42, 95-125. 10.2307/1942232

[JEB249319C52] Spray, D. C. (1974). Characteristics, specificity, and efferent control of frog cutaneous cold receptors. *J. Physiol*. 237, 15-38. 10.1113/jphysiol.1974.sp010467 4545023 PMC1350866

[JEB249319C53] Spray, D. C. (1986). Cutaneous temperature receptors. *Annu. Revi. Physiol*. 48, 625-638. 10.1146/annurev.ph.48.030186.003205 3085583

[JEB249319C54] Székely, D., Cogălniceanu, D., Székely, P. and Denoël, M. (2018). Dryness affects burrowing depth in a semi-fossorial amphibian. *J. Arid Environ*. 155, 79-81. 10.1016/j.jaridenv.2018.02.003

[JEB249319C55] Ultsch, G. R. (1989). Ecology and physiology of hibernation and overwintering among freshwater fishes, turtles, and snakes. *Biol. Rev*. 64, 435-515. 10.1111/j.1469-185X.1989.tb00683.x

[JEB249319C56] Vleck, D. (1981). Burrow structure and foraging costs in the fossorial rodent, *Thomomys bottae*. *Oecologia* 49, 391-396. 10.1007/BF00347605 28310003

[JEB249319C57] Wickham, H. (2016). *ggplot2: Elegant Graphics for Data Analysis*. New York, USA: Springer-Verlag.

[JEB249319C58] Windmiller, B. S. (1996). The pond, the forest, and the city: spotted salamander ecology and conservation in a human-dominated landscape. *PhD Thesis*, Tufts University, Boston, MA, USA.

[JEB249319C59] Wu, Q., Parker, S. L. and Thompson, M. B. (2009). Selected body temperature, metabolic rate and activity pattern of the Australian fossorial skink, *Saiphos equalis*. *Herpetol. J*. 19, 127-133.

