# Supplementary material for: Ups and downs of fossorial life: migration restlessness and geotaxis may explain overwintering emergence in the spotted salamander
Source: J Exp Biol. 2024 Oct 24;227(21):jeb249319. doi: 10.1242/jeb.249319 (PMC11529874; doi:10.1242/jeb.249319)
Supplement: Supplementary information [file jexbio-227-249319-s1.pdf]

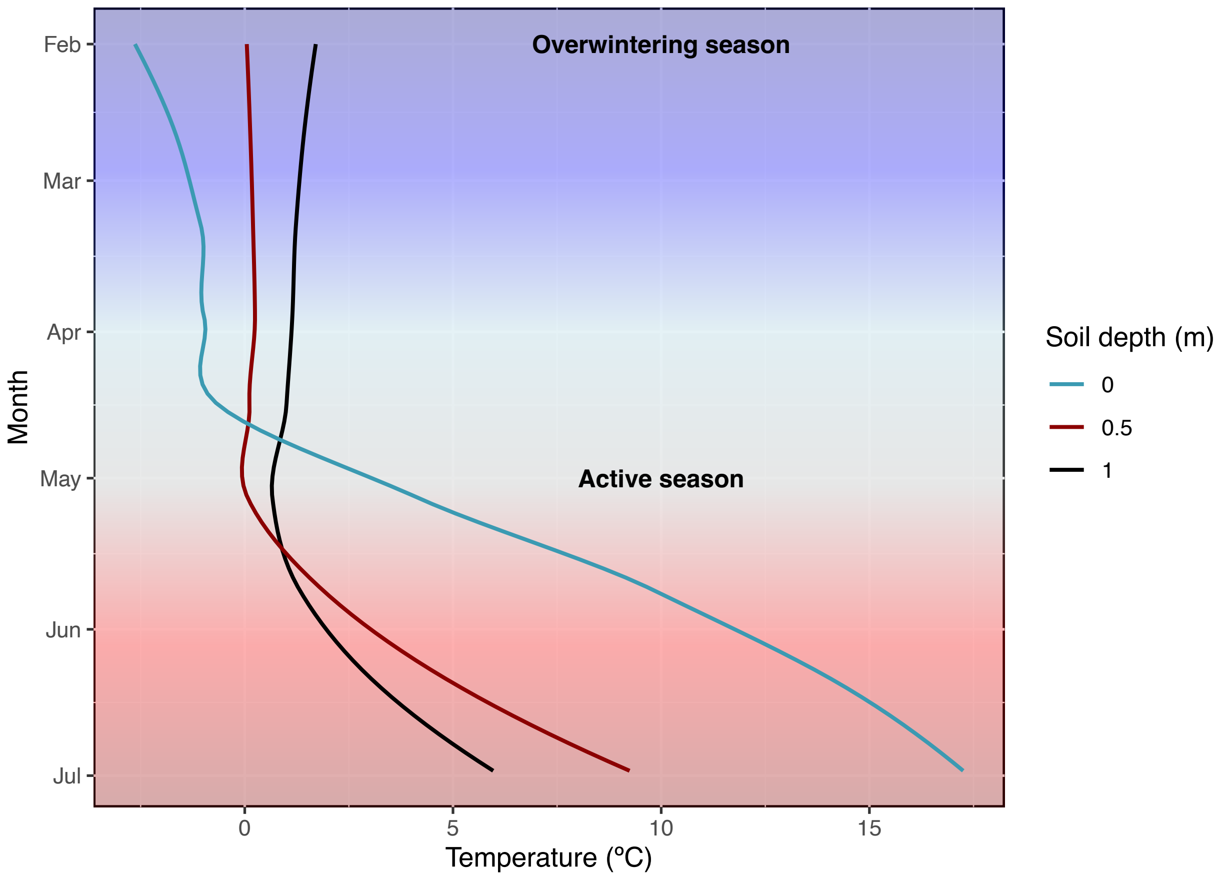

Fig. S1. Representation of soil temperature inversion in the forest surrounding Bat Lake, Algonquin Provincial Park, ON, Canada. Data were collected in 2018 using dataloggers (HOBO TidbiT® v2) set to record temperature every 2 h at three different soil depths (shallow = 0 m, intermediate = 0.5 m, deep = 1 m). During the winter, shallow depths are colder than deep ones. In the spring, the thermal gradient shifts and shallow depths become warmer than deep ones. Colour-coded lines show the predicted temperature at each soil depth in a given month. Annotations highlight the active (May–October) and overwintering (November–April) seasons. Raw data used with permission from Moldowan et al. (2022) The colour-coded background highlights the change in air temperature between seasons, ranging from cold (blue) to warm (red).

Journal of Experimental Biology • Supplementary information

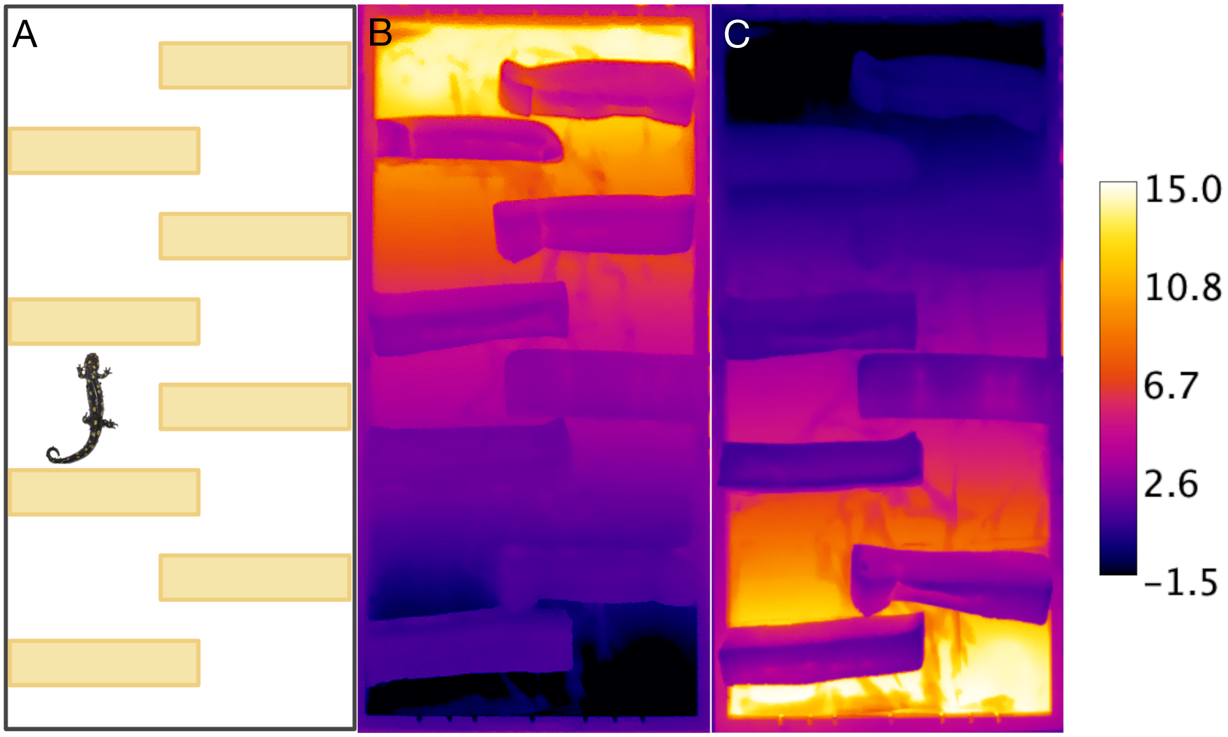

Fig. S2. A. Schematic of the vertical thermal gradient used to assess the effects of thermal inversion and gravity on salamander behaviour. Thermal image of the active (**B**) and overwintering (**C**) thermal gradients used in the study. The thermal gradient was always kept at a 45º angle relative to the horizontal axis, imitating underground burrows. Thermal gradient temperatures (°C) in **B** and **C** are colour-coded according to the temperature scale in the far-right panel.

Journal of Experimental Biology • Supplementary information

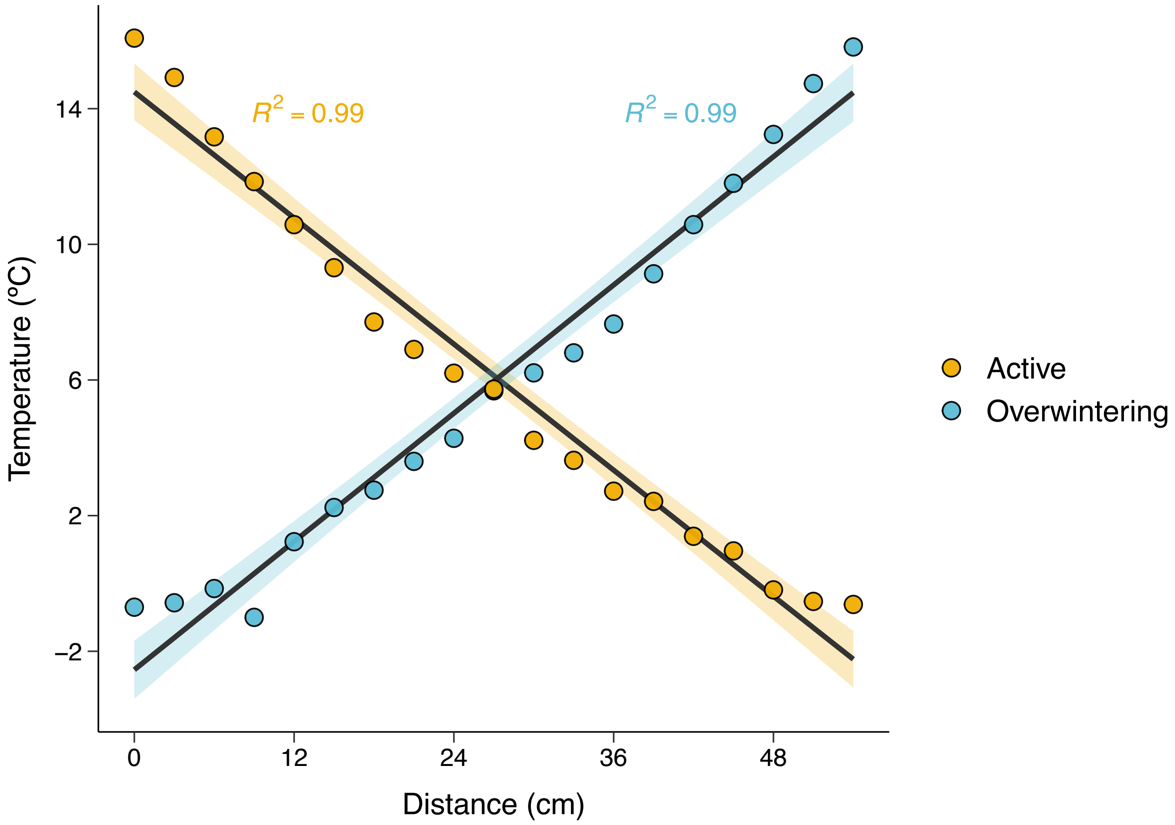

Fig. S3. The relationship between temperature and distance in the thermal gradient. Gradient temperature was measured every 3 cm from 0 cm (gradient top) to 54 cm (gradient bottom) under either thermal gradient orientation. Thermal gradient orientation and their respective R^2 values are colour-coded, with the active thermal gradient shown in orange and the overwintering thermal gradient shown in blue. For both thermal gradient orientations, the black solid lines and coloured shaded areas indicate the predicted relationship between temperature and gradient distance, and the 95% confidence interval, respectively.

Journal of Experimental Biology • Supplementary information

Table S1. Parameter estimates (β), 95% confidence intervals (95% CI), and *p*-values for the model testing how thermal gradient orientation affected median selected temperatures in *Ambystoma maculatum*. The model had median selected temperature as the response variable, sex and body mass as the predictors, and ID as a random term. Significant parameters are denoted in bold.

	**Median temperature ~ Gradient + Sex + Body mass + (1|ID)**		
Predictors	*Estimates*	*95% CI*	*p*
Intercept	7.54	3.06 – 12.02	**0.002**
Gradient (Overwintering)	-3.95	-5.43 – -2.47	**<0.001**
Sex (Male)	0.56	-1.64 – 2.75	0.605
Body mass	-0.09	-0.44 – 0.26	0.585
**Random Effects**			
σ^2	3.79		
τ_{00} Individual	0.94		
ICC	0.20		
N Individual	15		
Observations	30		
Marginal / Conditional R^2	0.482 / 0.585		

σ^2 = residual variance; τ_{00} Individual = individual variance; ICC = intraclass correlation coefficient; $N_{individual}$ = sample size.

Journal of Experimental Biology • Supplementary information

Table S2. Parameter estimates (β), 95% confidence intervals (95% CI), and *p*-values for the model testing how thermal gradient orientation affected total distance moved in *Ambystoma maculatum*. The model had total distance moved as the response variable, sex and body mass as the predictors, and ID as a random term. Significant parameters are denoted in bold.

		Total distance moved ~ Gradient + Sex + Body mass + (1|ID)		
Predictors		*Estimates*	*95% CI*	*p*
Intercept		16.81	-1.94 – 35.57	0.077
Gradient (Overwintering)		-7.78	-15.12 – -0.43	**0.039**
Sex (Male)		2.48	-6.64 – 11.59	0.580
Body mass		-0.68	-2.14 – 0.77	0.341
**Random Effects**				
σ^2	94.01			
$\tau_{00\ \text{Individual}}$	1.76			
ICC	0.02			
$N_{\text{Individual}}$	15			
Observations	30			
Marginal / Conditional R^2	0.217 / 0.231			

σ^2 = residual variance; $\tau_{00\ \text{Individual}}$ = individual variance; ICC = intraclass correlation coefficient; $N_{\text{individual}}$ = sample size.

Journal of Experimental Biology • Supplementary information

Table S3. Parameter estimates (β), 95% confidence intervals (95% CI), and *p*-values for the model testing how thermal gradient orientation affected normalised gradient position in *Ambystoma maculatum*. The model had normalised gradient position as the response variable and sex and body mass as the predictors. We did not include a random term in the test, because our linear mixed effects model had a singular fit. Significant parameters are denoted in bold.

	**Normalised gradient position ~ Gradient + Sex + Body mass**		
Predictors	*Estimates*	*95% CI*	*p*
Intercept	0.55	0.32 – 0.77	**<0.001**
Gradient (Overwintering)	0.06	-0.03 – 0.15	0.176
Sex (Male)	0.04	-0.07 – 0.15	0.474
Body mass	0.00	-0.02 – 0.02	0.868
Observations	30		
R^2	0.088		

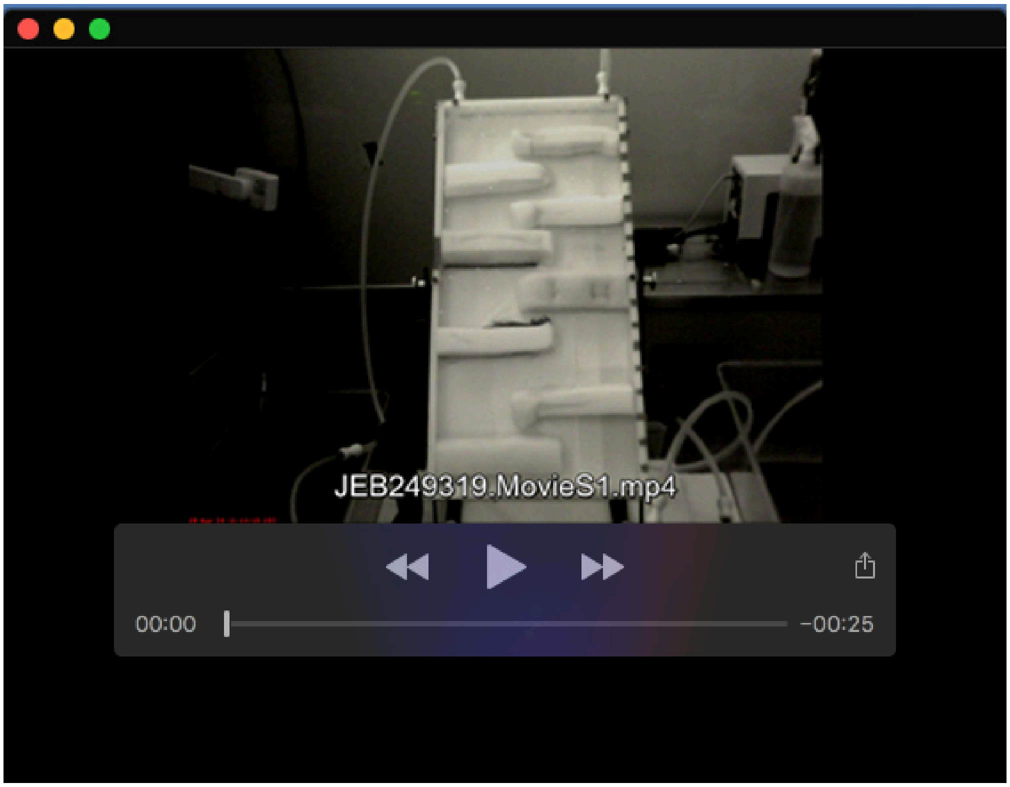

Movie 1. Sped-up time-lapse of a male *Ambystoma maculatum* tested within the active thermal gradient. Frames were taken every 30 s, for a total of 18 h of experiment (20 frames/sec).

Journal of Experimental Biology • Supplementary information
